# Could Arachidonic Acid-Derived Pro-Resolving Mediators Be a New Therapeutic Strategy for Asthma Therapy?

**DOI:** 10.3389/fimmu.2020.580598

**Published:** 2020-12-09

**Authors:** Daniella Bianchi Reis Insuela, Maximiliano Ruben Ferrero, Diego de Sá Coutinho, Marco Aurélio Martins, Vinicius Frias Carvalho

**Affiliations:** ^1^ Laboratory of Inflammation, Oswaldo Cruz Institute, Oswaldo Cruz Foundation (FIOCRUZ), Rio de Janeiro, Brazil; ^2^ Laboratory of Inflammation, National Institute of Science and Technology on Neuroimmunomodulation (INCT-NIM), Rio de Janeiro, Brazil

**Keywords:** asthma, lipoxins, PGE_2_, 15d-PGJ_2_, resolution

## Abstract

Asthma represents one of the leading chronic diseases worldwide and causes a high global burden of death and disability. In asthmatic patients, the exacerbation and chronification of the inflammatory response are often related to a failure in the resolution phase of inflammation. We reviewed the role of the main arachidonic acid (AA) specialized pro-resolving mediators (SPMs) in the resolution of chronic lung inflammation of asthmatics. AA is metabolized by two classes of enzymes, cyclooxygenases (COX), which produce prostaglandins (PGs) and thromboxanes, and lypoxygenases (LOX), which form leukotrienes and lipoxins (LXs). In asthma, two primary pro-resolving derived mediators from COXs are PGE_2_ and the cyclopentenone prostaglandin15-Deoxy-Delta-12,14-PGJ_2_ (15d-PGJ_2_) while from LOXs are the LXA_4_ and LXB_4_. In different models of asthma, PGE_2_, 15d-PGJ_2_, and LXs reduced lung inflammation and remodeling. Furthermore, these SPMs inhibited chemotaxis and function of several inflammatory cells involved in asthma pathogenesis, such as eosinophils, and presented an antiremodeling effect in airway epithelial, smooth muscle cells and fibroblasts *in vitro*. In addition, PGE_2_, 15d-PGJ_2_, and LXs are all able to induce macrophage reprogramming to an alternative M2 pro-resolving phenotype *in vitro* and *in vivo*. Although PGE_2_ and LXA_4_ showed some beneficial effects in asthmatic patients, there are limitations to their clinical use, since PGE_2_ caused side effects, while LXA_4_ presented low stability. Therefore, despite the strong evidence that these AA-derived SPMs induce resolution of both inflammatory response and tissue remodeling in asthma, safer and more stable analogs must be developed for further clinical investigation of their application in asthma treatment.

## Introduction

Asthma is a high prevalence chronic inflammatory pulmonary disease, the respiratory symptoms of which include cough, wheezing, shortness of breath, and chest tightness which leads to elevated morbidity, mortality, and disease social and economic costs (1–3). Pulmonary inflammation is the hallmark of asthma, which is driven by a Th2 immune reponse to inhaled allergens, and associated with infiltration of the bronchial mucosa with eosinophils, CD4+ T cells, macrophages and, in exacerbations and severe cases, neutrophils (4, 5). Macrophages are classified into classical (M1) or alternative activation (M2a, M2b, M2c, or M2d subtypes). During allergic asthma, under exposure to Th2 cytokines (IL-4 and IL-13), macrophages are reprogrammed to M2a profile and perform diverse functions ranging between protective and pathogenic roles (6–9). Airway remodelling is another key feature of asthma pathogenesis and can precede the development of inflammation (10). It is characterized by mucous gland and airway smooth muscle (ASM) cells hyperplasia and/or hypertrophy, deposition of extracellular matrix (EM) proteins, and myofibroblast proliferation, leading to the thickening and occlusions of airways (11). In severe asthmatic patients, a failure in pro-resolving pathways extends the pro-inflammatory mechanisms, resulting in a chronic inflammation, which is associated with a major cause of admission to the intensive care unit and high mortality rates (12, 13). Lipid mediators, such as those originated by arachidonic acid (AA), are key factors of the resolution of inflammation, once they orchestrate the clearance of pro-inflammatory cells and signals promoting tissue restoration (13, 14). In this review, we discussed the impact of AA-derived specialized pro-resolving mediators (SPMs) in the resolution of inflammation and remodeling in asthma.

## Resolution of Inflammation

The resolution of inflammation is an active and controlled process that reduces inflammation through the elimination of danger signals, leading to the restoration of tissue homeostasis and preventing the progression towards an uncontrolled chronic inflammatory state. Thus, catabolization and antagonization of pro-inflammatory mediators, a decrease in leukocyte numbers at inflammatory sites, and tissue repair are key events in the resolution process (13). It is notewothy that different from classical anti‐inflammatory molecules, the SPMs modulate the end of the inflammatory response, without promoting unwanted immunosuppression (15). During the resolution phase, leukocyte apoptosis and metabolization of intracellular inflammatory signals lead to the clearance of inflammatory cells by specialized phagocytes. Together, these events promote the end of the acute inflammatory response and initiate tissue repair and healing (12, 16).

Endogenous mediators that actively participate in the resolution process include lipids (i.e., lipoxins, resolvins, maresin, and protectins), peptides (i.e. alpha-melanocortin-stimulating hormone and chemerin), proteins (i.e., annexin A1, Galectin-1, TGF-β and IL-10), and nucleotides (i.e. adenosine and inosine) (17–19). They promote cessation of polymorphonuclear infiltration into the inflamed tissue, reprogramming of macrophages and TCD4^+^ cells to M2 and T regulatory phenotypes respectively, sequestration and counter-regulation of pro-inflammatory mediators, apoptosis of polymorphonuclear cells with subsequent phagocytosis by M2 macrophages, and tissue repair (20–22).

Among the SPMs, the lipid mediators activate many aspects of the resolution process (23). These endogenous mediators are biosynthesized in local inflamed tissue microenvironments, and can control the magnitude/duration of the inflammatory response as well as the timing of tissue restoration (17). They primarily come from the metabolism of polyunsaturated fatty acids, such as AA, docosahexaenoic acid (DHA), eicosapentaenoic acid (EPA), and docosapentaenoic acid (DPA) (15). Interestingly, several AA-derived mediators have consistently presented pro-resolving and tissue protecting activities in asthma (24, 25).

## SPMs Derived From Cyclooxygenases

Cyclooxygenases **(**COX), especially COX-2 isoform, play a pivotal role in the conversion of AA into different pro-inflammatory lipid mediators, including prostaglandins (PG) and thromboxanes (26). Despite the clear ligation of COX-2 activity with the development of the inflammatory response, it has also been proved that the inhibition of this enzyme impairs leukocyte clearance, indicating that some COX-2 derived mediators possess pro-resolving action. This occurs mainly due to the ability of COX-2 to metabolize EPA into resolvins, which are one of the main classes of SPMs (27). In asthma, beyond resolvins, the COX-2 activity also culminates in the formation of other important SPMs, such as PGE_2_ and 15-Deoxy-Delta-12,14-PGJ_2_ (15d-PGJ_2_), a metabolite of PGD_2_ (28).

PGE_2_ is synthesized by three distinct enzymes, microsomal PGE synthase-1 (mPGES-1), mPGES-2, and cytosolic PGES (cPGES), which use PGH_2_ as substrate. The actions of PGE_2_ are mediated by four distinct 7TM receptors (EP1–EP4) (29). Although PGE_2_ is a pro-inflammatory mediator, several works have shown that this lipid presents pro-resolving actions in some contexts (30). So, what determines when PGE_2_ presents pro-resolving effects? There are three major factors, not mutually exclusive: i) time: the kinetics of PGE_2_ release can separate its pro-inflammatory and pro-resolving effects due to the presence of different targets (31, 32); ii) context: eg. PGE_2_ can inhibit ERK activation and MMP-1 secretion by gastric epithelial cells in the presence of cytokines, however, in their absence, PGE_2_ does the opposite (31, 33, 34); iii) concentration: eg. very low PGE_2_ concentrations inhibit chondrocyte-dependent collagen cleavage in osteoarthritis cartilage, while higher concentrations enhance it (35).

IL-4 and IL-13, essential cytokines in the asthma pathogenesis (4), suppressed PGE_2_ production by dendritic cells through reduction of COX-2 and mPGES-1 expression (36). Also, asthmatic patients presented an inverse correlation between the sputum levels of PGE_2_ and eosinophil numbers (37, 38), suggesting that PGE_2_ may reduce airway eosinophilia in these patients. Inhaled PGE_2_ markedly inhibits the early and late bronchoconstrictor response to an allergen in asthmatic patients (39); however, these effects may be related only to the PGE_2_-induced ASM relaxation (40). Nevertheless, COX-1 knock out (KO) and EP2KO mice that were ovalbumin (OVA)-sensitized and challenged showed increased eosinophilia and Th2 cytokines levels in the lungs and bronchoalveolar lavage (BAL), respectively, compared to wild-type (WT) mice (41, 42). Besides, treatment with PGE_2_ inhibited the house dust mite (HDM)-induced lung eosinophilia (43), and OVA-provoked accumulation of eosinophils and Th2 cytokines in the BAL (42), probably because PGE_2_ can inhibit β_2_ integrin and L-selectin function with a consequent reduction in eosinophil migration (44, 45). Furthermore, prior studies of our group showed that PGE_2_ derived from eosinophils induced an early resolution of allergic pleural edema (25, 46).

Until now, there has been no agreement on the effects of PGE_2_ on the differentiation of naïve T cells to Th1, Th2, or Th17 (47); however, type 2 innate lymphoid cells (ILC2), that emerged in the literature as novel Th2 cytokine-producing cells, strongly express both EP2 and EP4. PGE_2_ inhibited proliferation, activation, and release of cytokines by ILC2 (48, 49). Besides, alveolar macrophages from asthmatics presented a reduction in the EP2 expression (50) and PGE_2_ generation, in parallel with decreased efferocytosis of apoptotic cells (51). PGE_2_ is a well-known inductor of M2 macrophage reprogramming (52). Furthermore, PGE_2_ induced IL-10 production by macrophages *in vitro*, and the adoptive transfer of those PGE_2_‐treated macrophages led to fewer infiltrating eosinophils, macrophages, activated TCD4+, and regulatory T lymphocytes in lungs of HDM‐exposed mice (43).

In lung fibroblasts, there is an inverse relationship between COX-2 and mPGES-1 expression and the number of allergen challenges, resulting in a reduction in PGE_2_ production by those cells (53). Besides, mPGES-1 KO mice showed an augmentation of allergen-induced vascular smooth muscle cell numbers and thickness of intrapulmonary vessels (54). PGE_2_ also inhibited fibroblast migration, proliferation, collagen deposition, and myofibroblast differentiation in the lung (55). *In vitro*, PGE_2_ decreased the expression of tenacin C and fibronectin by human fibroblast and ASM cells (56), reduced the proliferation of ASM cells derived from asthmatic patients (57), and upregulated the expression of the anti-inflammatory protein tristetraprolin in human ASM cells (58). Prior investigations of our group revealed that the instillation of glucagon induced a high production of PGE_2_ into the lungs (59). Also, we reported that a non-selective COX inhibitor decreased the inhibitory effect of glucagon on OVA-induced collagen deposition in the lungs (60), suggesting that the anti-remodeling effect of glucagon depends on PGE_2_ production. Interestingly, inhaled PGE_2_ showed bronchodilator capacity in small clinical trials with asthmatic patients (61, 62) (**Table 1**).

**Table 1:** Summary of clinical studies using mediators related to arachidonic acid metabolism pathways in asthma.

Drug	Classification	Key Results	Side Effects	Ref.
PGE_2_	PGE_2_	Inhalation of PGE_2_ inhibited the early and late bronchoconstriction response to inhaled allergen in asthmatic patients	Cough and retrosternal soreness transient	(39)
PGE_2_	PGE_2_	Inhalation of PGE_2_ reduced exercise-induced bronchoconstriction in asthmatic patients	Cough and retrosternal soreness transient	(61)
PGE_2_	PGE_2_	Aerosolization of PGE_2_ had a bronchodilator effect in patients with bronchial asthma	Headache, cough and irritation of the pharynx	(62)
Indomethacin	COX inhibitor	Oral administration of Indomethacin induced a slight decrease in allergy sensitivity measured by specific airway conductance in asthmatic patients	No side effects were evaluated	(63)
Indomethacin	COX inhibitor	Inhalation of Indomethacin reduced exercise-induced bronchoconstriction in asthmatic children	No side effects were evaluated	(64)
Etoricoxib	COX-2 inhibitor	Etoricoxib had no effect on allergen-induced airflow obstruction and sputum eosinophils, basal lung function, or methacholine responsiveness in mild asthma patients	No side effects were observed	(65)
Pioglitazone	PPAR-γ agonist	Pioglitazone had no effect on symptoms, airflow obstruction and inflammation in patients with severe asthma	Peripheral edema and presumptive angioedema	(66)
LXA_4_	LXA_4_	Nebulization of LXA_4_ inhibited LTC4-induced airway obstruction in asthmatic patients	No side effects were observed	(67)
5(S),6(R)-LXA_4_ methyl ester	LXA_4_ analog	Inhalation of 5(S),6(R)-LXA_4_ methyl ester improved pulmonary function in asthmatic children with acute episodes	No side effects were observed	(68)
BML-111	LXA_4_ receptor agonist	Inhalation of BML-111 improved pulmonary function in asthmatic children with acute episodes	No side effects were observed	(68)

COX, Cyclooxygenase; LXA_4_, Lipoxin A_4_; PGE_2_, Prostaglandin E_2_; PPAR-γ, Peroxisome proliferator-activated receptor; Ref, References.

Despite the possible benefits of PGE_2_ in asthma, non-selective COX inhibitors improved specific airway conductance and airway constriction of asthmatics (63, 64), suggesting that COX-derivatives may play a role in the development or worsening of asthma. Nevertheless, patients with mild allergic asthma treated with specific COX-2 inhibitors did not present an effect on lung function and eosinophil accumulation in the sputum (65) (**Table 1**). Although PGE_2_ acts directly in the resolution of inflammation, it can also drive a pro-inflammatory response in human fibroblast and ASM cells (56). Furthermore, PGE_2_ apparently desensitized β2 adrenergic receptors during asthma exacerbation triggered by Rhinovirus infection (69). A high dose of PGE_2_ can also induce airway contraction in asthmatic patients, probably through activating different receptors (70), and cough by activation of EP3 receptor (71). As the most of pro-resolving actions of PGE_2_ are related to the activation of EP2, the development of selective agonists of this receptor can be a good strategy to be consider for treating asthma in the future.

15d-PGJ_2_ is formed spontaneously by a series of dehydration of PGD_2_ (72), and it is produced abundantly in the inflamed site, making it important in the resolution of the inflammation (73). Most of the pro-resolving actions of 15d-PGJ_2_ depend on the peroxisome proliferator-activated receptor-gamma (PPARγ) activation, but some of its effects are independent of this receptor (74). In asthmatic patients, there is a reduction in the PPARγ expression in BAL cells (75). Furthermore, polymorphism of the PPARG gene may be related to an increased risk of asthma development (76). Activation of PPARγ by synthetic agonists reduced the levels of Th2 cytokines and inhibited AHR, the influx of eosinophils and structural changes in the airway wall in murine OVA-challenge models of asthma (77, 78). Together, these data indicate that the reduction in PPARγ expression by inflammatory cells in asthmatic patients may be one of the mechanisms that contribute to the development of chronic asthma.

In a model of carrageenin-induced pleurisy, 48h after the provocation, when mononuclear cells dominate the reaction up to the resolution, there was an immense increase in COX-2 protein expression and 15d-PGJ_2_ levels coincident with inflammatory resolution and associated with minimal exudate PGE_2_ levels. In this model, the use of both nonselective or selective COX-2 inhibitors, 24h after carrageenin challenge, increased the number of inflammatory cells and exudate volume in parallel to a reduction in the 15d-PGJ_2_ levels. In addition, 15d-PGJ_2_ reversed the selective-COX-2 inhibitor-induced rise in cell number and exudate volume, indicating that the production of 15d-PGJ_2_ is important to the resolution in this model (79). The pro-resolving effect of 15d-PGJ_2_ was related to an induction of apoptosis of inflammatory cells (80). Besides, 15d-PGJ_2_ also regulates the balance of cytokines and chemokines that control leukocyte trafficking during acute inflammation, promotes M2 macrophage differentiation, as well as the efflux of macrophage to draining lymphatics, facilitating the resolution of inflammation (81). This pro-resolving effect of 15d-PGJ_2_ may be dependent on PPARγ, once IL-4-induced PPARγ activity becomes indispensible for M2 activation (82, 83).

In an OVA-induced asthma model, KO mice for PGD synthase (H-PGDS), an enzyme that catalyzes PGH_2_ into PGD_2_, showed accelerated chronic allergic lung eosinophil inflammation in parallel to an increase in the local levels of TNFα and eotaxin-1. Furthermore, the exogenous administration of 15d-PGJ_2_ decreased the excessive eosinophilic infiltration and TNFα and eotaxin-1 levels noted in those mice (84). Furthermore, the activation of PPARγ reduced OVA-induced eosinophilia and IL-4, IL-5, and IL-6 levels in the lungs of mice (85). We previously showed that interventional treatment with 15d-PGJ_2_ inhibited both OVA- and HDM-induced eosinophils accumulation and IL-5 and IL-13 levels in the lungs (86). The pro-resolving effect of 15d-PGJ_2_ on lung eosinophilia is probably related to its ability to block the traffic and induce apoptosis of these granulocytes (87). The inhibitory effect of 15d-PGJ_2_ on eosinophil migration is possibly dependent on PPARγ, once the activation of this receptor by synthetic agonists inhibits chemotaxis of eosinophils (85). However, the pro-apoptotic effect of 15d-PGJ_2_ is independent of PPARγ (87). 15d-PGJ_2_ also inhibited T lymphocyte proliferation in a mechanism probably dependent on PPARγ, as it is mimicked by PPARγ synthetic agonists (88, 89).

We previously demonstrated that interventional treatment with 15d-PGJ_2_ reversed structural changes related to airway remodeling, including epithelial thickening, mucus exacerbation, and EM deposition, in both OVA and HDM murine models of asthma (86). These antiremodeling effects of 15d-PGJ_2_ may be related to its ability to reduce differentiation of fibroblasts into myofibroblasts, the proliferation of myofibroblasts (90), and fibroblast growth factor-induced human ASM cell proliferation (91). Although PPARγ agonists are extremely promising to asthma therapy, unfortunately severe asthmatic patients treated with pioglitazone did not present with an improvement in asthma features and showed significant side effects (66) (**Table 1**).

## SPMs Derived From Lipoxygenases

5- Lipoxygenase (LOX) and 15-LOX are the main LOXs involved in the metabolization of AA (92), resulting in the formation leukotrienes (LTs) and lipoxins (LXs). While LTs are recognized to exert broad proinflammatory effects, LXs present pro-resolving actions (93). Endogenously, LXs are typically produced by three main pathways. In one route, LXA_4_ and LXB_4_ are produced by 5-LOX (94), and in other by 12-LOX (95). It is described that aspirin treatment can also promote the synthesis of LXs epimers denominated aspirin-triggered lipoxins (ATLs), including 15-epimeric (epi)-LXA_4_ and 15-epi-LXB_4_ (94). LXA_4_ and ATLs act primarily on a 7TMN receptor denominated ALXR (96). ALXR is expressed in several tissues, including lungs, and different cell types such as leukocytes, fibroblasts, and bronchial epithelial cells. LXA_4_ can also activate the aryl hydrocarbon receptor, and both LXA_4_ and ATLs are antagonists of the cysteinyl leukotriene receptor 1 (97). Nevertheless, the LXB_4_ receptor has not yet been identified (98).

The failure in the generation and action of LXs is associated with more severe airway inflammation (99). Indeed, severely asthmatic patients presented a reduction of LXA_4_ levels in BAL fluid, sputum, and whole blood compared to moderately asthmatic individuals. This reduction in LXA_4_ concentrations observed in severe asthma was associated with a higher degree of airway obstruction (24). Eosinophils from the blood of asthmatic patients presented a decreased ALXR expression compared to those obtained from healthy individuals (100). Furthermore, transgenic mice that overexpress ALXR showed a reduction in OVA-induced eosinophilia in the BAL and lung tissue (101). We previously showed that 15-epi-LXA_4_ analogs inhibited OVA-induced pleural eosinophil influx by reducting local eotaxin and IL-5 generation (102). We also noted that 15-epi-LXA_4_ analogs accelerate the drainage of OVA-induced pleural edema (25). In human eosinophils, LXA_4_ inhibited chemotaxis toward chemoattractants (103), and granulocyte-macrophage colony-stimulating factor-induced IL-13 and eotaxin release *in vitro* (104). In spite of inhibiting eosinophil migration, 15-epi-LXA_4_ is a potent chemoattractant to monocytes *in vitro* (105) and restored the balance between M2 and M1 populations into the lungs in a murine model of pulmonary damage induced by bleomycin (106). Furthermore, LXA_4_ stimulates macrophage efferocytosis of apoptotic polymorphonuclear cells and cellular debris (107).

Among the ILC family, natural killer (NK) cells and ILC2s are important in the control and exacerbation of asthma, respectively. NK cell depletion induced a persistent allergic airway inflammation in association with reduction of the LXA_4_ levels in the BAL (108). LXA_4_ enhanced activated NK cells-induced eosinophil apoptosis through ALXR activation (109). Meanwhile, the blood and sputum of patients with severe asthma presented elevated numbers of ILC2 compared to mild asthmatics, which was related to persistent airway eosinophilia (110). LXA_4_ inhibited both PGD_2_- and IL-25 plus IL-33-induced IL-13 release by ILC2 *in vitro* in a mechanism dependent on ALXR activation (109).

*In vitro*, LXA_4_ and 15-epi-LXA_4_ reduced IL-8 secretion induced by serum amyloid A in a human alveolar A549 cell line (111). Also, activation of ALXR by LXA_4_ increased basal proliferation and wound repair of human airway epithelial cells (112). In a murine model of asthma caused by OVA, airway epithelial cells presented with an increased expression of ALXR (101) and LXB_4_ reduced mucus production (113). ASM hypertrophy and hyperplasia, as well as accumulation of muscle cells in the subepithelial layer, are some of the changes observed in asthma remodeling. It was shown that LXA_4_ reduced both LTE_4_- and IL-13–primed ASM migration toward platelet-derived growth factor *in vitro* (114). Another critical pathological feature of airway remodeling in asthma is the EM deposition in the peribronchiolar area. It is noteworthy that both fibroblasts and myofibroblasts can express ALXR (115). Moreover, LXA_4_ inhibited connective tissue growth factor-induced human lung fibroblast proliferation *in vitro* (115) and blocked TGF-β-triggered increase in α-smooth muscle actin expression and collagen release by human myofibroblasts *in vitro* (116). Besides, treatment with 15-epi-LXA_4_ reversed bleomycin-promoted fibrosis and lung damage in mice (106). Altogether, these data suggest a potential role of LXs in the resolution of the airway and peribronchiolar remodeling observed in asthmatics.

Due to the possible therapeutic application of LXA_4_, some clinical trials using this LX, its analogues, or LXA_4_ receptor agonist BML-111 were administered in asthmatic patients. The nebulization of LXA_4_ reduced LTC4-induced bronchoconstriction (67); however, the rapid inactivation and significant instability to exposure to light and acids of LXA_4_ (117) make its clinical use difficult. Furthermore, the inhalation of LXA_4_ analog or BML-111, which is more potent and stable than LXA_4_ itself (118), improved the lung function (68) (**Table 1**). Interestingly, both LXA_4_ analog and BML-111 were well tolerated and presented no side effects (68).

## Conclusion

**Figure 1:**
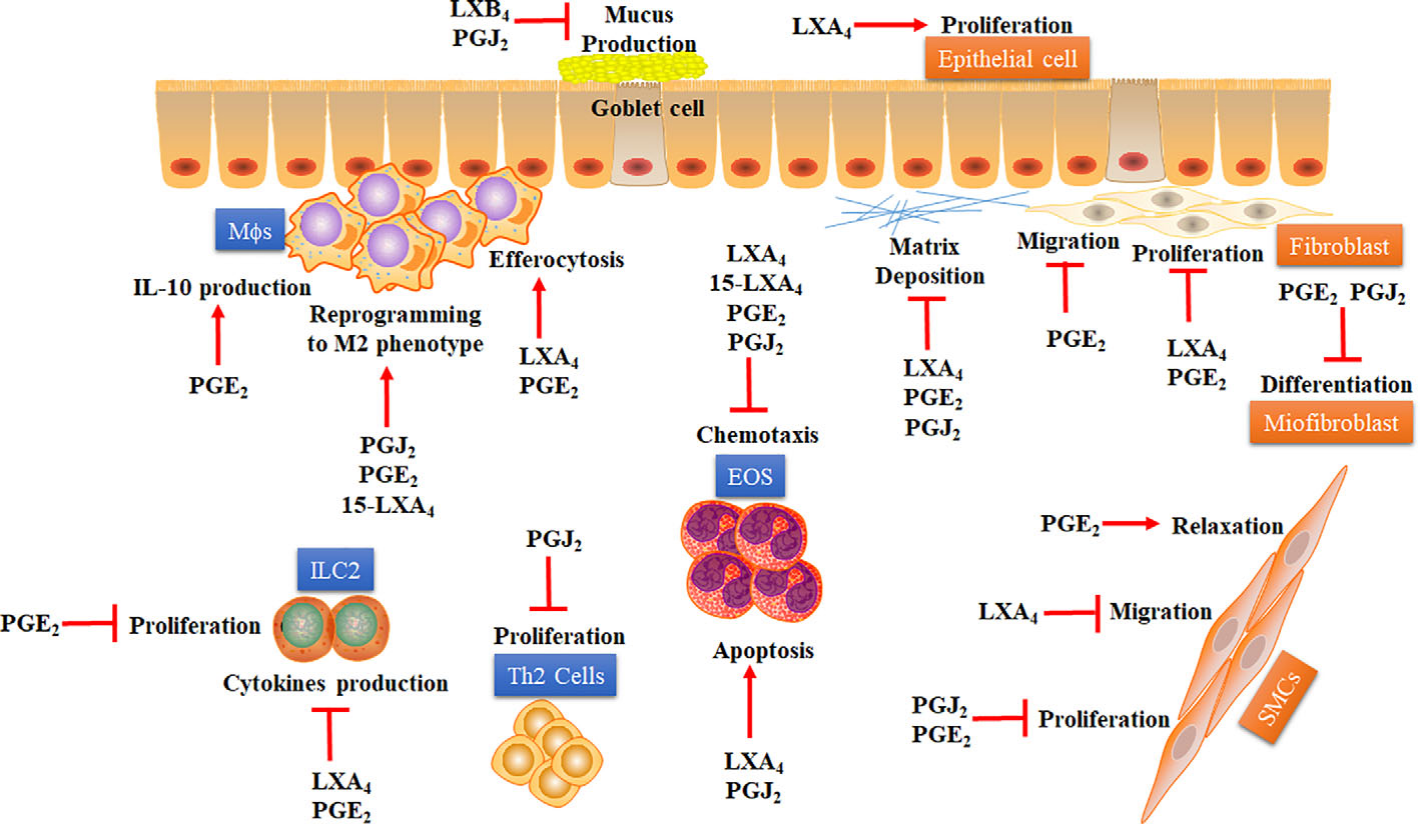
Proposed mechanisms whereby COX- and LOX-derived lipid mediators may accelerate the resolution of lung inflammation in asthma. Some COX- and LOX-derived lipid mediators, including PGE_2_, 15dPGJ2, LXA_4_, and LXB_4_, have demonstrated several pro-resolving actions over immune cells (blue squares) and structural cells (orange squares) involved in asthma. Pro-resolving effects of COX- and LOX-derived lipid mediators are: i) inhibition of EOS chemotaxis and stimulation of apoptosis on those cells; ii) inhibition of ILC-2 proliferation and cytokine production; iii) inhibition of Th2 lymphocytes proliferation; iv) stimulation of efferocytosis and IL-10 production by MΦs; v) induction of macrophage reprogramming to alternative M2 phenotype. Besides, these SPMs derived from COX and LOX present some important antiremodeling effects in asthma, like: i) inhibition of mucus production by globet cells and stimulation of airway epithelial cells proliferation; ii) inhibition of proliferation and migration of SMCs and stimulation of relaxation of these cells; iii) inhibition of proliferation, migration, and extracellular matrix deposition by fibroblasts; iv) inhibition of fibroblast differentiation into myofibroblasts. EOS: Eosinophil. ILC-2: Type-2 innate lymphoid cells. LXA_4:_ Lipoxin A_4_. 15-LXA_4_: 15-epimeric (epi)-LXA_4_. LXB_4_: Lipoxin B_4_. MΦs: Macrophages. M2: M2 macrophage phenotype. PGE_2_: Prostaglandin E_2_. PGJ_2:_ 15-Deoxy-Delta-12,14-PGJ_2_. SMCs: Smooth muscle cells. Th2: Type-2 CD4+ T helper. The arrow represents stimulation while the flat arrow represents inhibition.

This mini-review presents several aspects of the pro-resolving effects of COX- and LOX-derivative mediators in asthma (**Figure 1**), addressing their efficacy and current limitations for clinical use. Nevertheless, the review presents several strong pieces of evidence that support the development of new drugs based on analogs of PGE_2_, 15d-PGJ_2_, and LXs with better physical-chemical properties, allowing greater stability and superior selectivity for specific receptors. Moreover, new analogs of AA-derived SPMs could also improve efficiency and reduce the required dose of glucocorticoid, the latter often leading to adverse effects and steroid-refractoriness, despite being the best asthma treatment so far.

## Author Contributions

DI, MF, and DC contributed to the conception and design of the study, wrote the manuscript, discussed the content, and contributed to the manuscript revision. MM discussed the content and contributed to the manuscript revision. VC contributed to the conception and design of the study, wrote the manuscript, discussed the content, and contributed to the manuscript revision. All authors reviewed and/or edited the manuscript prior submission. All authors contributed to the article and approved the submitted version.

## Funding

This work was supported by PrInt Fiocruz-CAPES Program N° 01/2020.

## Conflict of Interest

The authors declare that the research was conducted in the absence of any commercial or financial relationships that could be construed as a potential conflict of interest.

## Abbreviations

AA, arachidonic acid; AHR, airway hyperreactivity; ASM, airway smooth muscle; ATLs, aspirin-triggered-lipoxins; BAL, bronchoalveolar lavage; COX, cyclooxygenase; cPGES, cytosolic PGE synthase; DHA, docosahexaenoic acid; DPA, docosapentaenoic acid; 15d-PGJ_2_, 15-deoxy-delta-12,14-PGJ_2_; EM, extracellular matrix; EPA, eicosapentaenoic acid; Epi, epimeric; HDM, house dust mite; H-PGDS, PGD synthase; ILC2, type 2 innate lymphoid cells; KO, knock out; LOXs, lipoxygenases; LTs, leukotrienes; LXs, lipoxins; mPGES-1, microsomal PGE synthase-1; NK, natural killer; OVA, ovalbumin; PG, prostaglandin; PPAR-γ, peroxisome proliferator-activated receptor; SPM, specialized pro-resolving mediator; WT, wild-type.
